# Characteristics of Youth Crisis App Users: Mental Health Service Access and Barriers and Perceptions of Helpfulness

**DOI:** 10.1016/j.jaacop.2024.06.006

**Published:** 2024-08-28

**Authors:** Mindy Westlund Schreiner, Brian W. Farstead, Myah Pazdera, Amanda V. Bakian, Brent M. Kious, Karen Manotas, Sheila E. Crowell, Erin A. Kaufman, Scott A. Langenecker

**Affiliations:** a Nationwide Children’s Hospital, Columbus, Ohio; b Ohio State University, Columbus, Ohio; c University of Utah, Salt Lake City, Utah; d University of Oregon, Eugene, Oregon

**Keywords:** crisis, barriers, self-injurious thoughts and behavior, technology, youth

## Abstract

**Objective:**

By developing a more nuanced understanding of youth using crisis line services, greater strides can be made in addressing their mental health needs. SafeUT is an app-based mental health crisis service that is offered to nearly all youth living in Utah and provides 24/7 access to licensed clinicians by phone or text. This study characterized youth using a statewide mental health crisis service and identified barriers to care.

**Method:**

SafeUT users were offered the opportunity to complete a survey regarding demographics, their experiences with mental health services, and self-injurious thoughts and behaviors.

**Results:**

A total of 210 youths completed at least part of the survey. More than half of the sample identified as LGBTQ+ (lesbian, gay, bisexual, transgender, queer, and others). Less than half of the sample had received mental health services. Not wanting to talk to a parent/guardian was the most frequently endorsed barrier. In the 2 weeks preceding the survey, 50% of youth engaged in self-injurious behaviors (18% suicide attempts). Following their SafeUT encounter, youth indicated significant reductions in the intensity of their presenting concerns.

**Conclusion:**

SafeUT appears to be effective in reducing acute concerns. However, youth still receive insufficient mental health support. Addressing barriers is imperative in ensuring that youth receive appropriate mental health care.

## Plain language summary

Youth mental health crisis app users were asked to complete a survey after completing an encounter with an app clinician. About half of respondents reported engaging in self-harm in the past two weeks. Less than half reported receiving formal mental health services, with not wanting to talk to a parent or guardian being the most endorsed barrier (54%). While a significant decrease in the intensity of presenting concerns followed app interaction, more work is needed to address barriers to additional community mental health services for these high-risk youth.

There is promising evidence that mental health crisis services for suicide prevention, including services deployed on a national level, are effective.^1^ Prior research on the effectiveness of these programs, specifically Australia’s National Youth Suicide Prevention Strategy, found that adolescents reported significant improvements in mental state and reductions in suicidality following their encounter relative to immediately before their crisis line encounter.^2^ Studies of telephone crisis lines in the United States have reported that callers experienced a reduction in psychological pain and hopelessness in the weeks following their contact with the crisis line.^3^ Despite these encouraging findings, research closely examining who uses and benefits from mental health crisis services is sparse, especially in youth. This is a particularly important time to investigate these questions, given the rollout of the 988 Suicide and Crisis line in 2022, the vast number of individuals who use the crisis line or similar crisis services,^4^ and the paucity of outcomes data available. Additionally, extant research to date on even the short-term outcomes of crisis line usage is often limited to callers as opposed to texters or individuals using similar chat options. In a recent study of the Crisis Text Line in the United States, 90% of texters struggling with suicidality indicated that their text exchange was helpful, with nearly half endorsing feeling less suicidal.^5^

In response to suicide being the leading cause of death for youth ages 10 to 24 in Utah, the Utah State Legislature created a commission that led to the development of the SafeUT crisis text app service. SafeUT is a crisis service that offers anonymous support via text-based chat or phone call. Users can also provide anonymous tips regarding concerns they may have about bullying, violence, drugs, and other issues, which are then routed to appropriate school officials. This service first became available in early 2016 as a free app to students (elementary through college), their educators, and their parents/guardians, and it currently is available to more than 98% of youth in Utah through their educational environments. The availability of SafeUT was also expanded to Utah National Guard members in 2019 and frontline workers (police, firefighters, emergency medical personnel, and health care providers) in 2020. While there are many similarities to the National Suicide Hotline, such as 24/7 availability and the option to either text or call, SafeUT provides a higher level of care: there is access to licensed clinicians who respond to all incoming concerns by providing supportive or crisis counseling and referral services, whereas other services tend to use nonclinical volunteers. In some cases, the clinician may opt to deploy a mobile crisis outreach team or, if unavailable, dispatch emergency service teams.^6^ SafeUT also differs from other services in its integration with the schools, including bidirectional communication. Users can submit tips regarding concerns including mental health, firearms, and drug use to SafeUT, which is then triaged to the appropriate school personnel. Additionally, if a SafeUT user is experiencing an active suicidal crisis in need of intervention, SafeUT can interact with the school associated with the user’s profile in an effort to obtain needed contact information to ensure safety. Finally, SafeUT allows for the development of profiles for frequent users (with permission), which can facilitate rapport building and personalization of crisis management strategies.

The present study provides initial descriptive information regarding the characteristics of SafeUT crisis text line users and their perceptions of the effectiveness of the app. We were also interested in the experiences SafeUT users had with other mental health services as well as barriers they encountered in trying to access care. Attaining a better understanding of the characteristics of crisis resource users could facilitate better-tailored responses on the part of clinicians and crisis service volunteers and improve outcomes as well as development of new services for these youth.

## Method

### Ethics and Recruitment

This study was approved by the institutional review board and underwent an ethics consultation through the Office of Research Integrity and Compliance at the University of Utah. Because maintaining anonymity for SafeUT users was a major priority, we sought and received a waiver of parental consent from the institutional review board. In the year before the survey going live in August 2021, the median number of messages within each chat was 26. Based on this information and the desire to have the survey completed by app users who had a reasonably clear interaction of sufficient intensity with a SafeUT clinician, we offered the survey to 1 in 5 SafeUT users who had greater than 10 exchanges (or volleys, which are back-and-forth chats between user and clinician) between August 2021 and the end of October 2022. This opportunity was presented immediately following the conclusion of their chat with a SafeUT clinician. Assent/consent and surveys were delivered using REDCap hosted at the University of Utah.^7^^,^^8^ Before beginning the first survey, participants were provided with information regarding the potential risks and benefits of the study. Participants provided their e-mail addresses because this was a longitudinal study with compensation in the form of gift cards. Providing their e-mail after they had reviewed the study information indicated their consent to participate in the study and provided a means by which we could compensate them and provide follow-up surveys. Participants then completed a survey, which is included in Supplement 1, available online.

### Survey Content

Participants were asked to select barriers they have encountered in trying to access mental health service from a list of items (see Supplement 1, available online). Participants were asked to rate the level of difficulty for each barrier they endorsed on a scale of 1 (“not at all difficult”) to 5 (“extremely difficult”). Participants were asked about their experiences with self-injurious thoughts and behaviors (SITBs) in the 2 weeks before completing the survey. They were asked whether they had specific SITB experiences, and for each experience endorsed, participants rated the intensity and frequency of their experience on a scale of 1 (“not at all”) to 5 (“very”). Participants were asked about the presenting concerns experienced immediately before their SafeUT encounter and survey completion. For each concern endorsed, participants were prompted to rate the intensity of this experience before and after their SafeUT encounter. Intensity ratings were on a scale of 1 (“not at all intense”) to 5 (“very intense”).

Participants were asked to rate how supported they felt by their SafeUT counselor, how satisfied they were with how their concern was handled, how often they felt the counselor listened carefully to them, how often they felt the counselor explained things in a way they could understand, and how often they felt the counselor showed respect for what they had to say. For support, options included “very unsupported,” “somewhat supported,” “neither unsupported nor supported,” “somewhat supported,” “very supported,” and “unsure.” Regarding satisfaction, options included “very dissatisfied,” “somewhat dissatisfied,” “neither satisfied nor dissatisfied,” “somewhat satisfied,” “very satisfied,” and “unsure.” On the remaining items assessing satisfaction (how often counselor listened carefully, explained things well, and showed respect), options included “never,” “rarely,” “sometimes,” “usually,” and “always.”

### Data Analyses

Before completing statistical analyses in R,^9^ data were carefully examined to remove duplicate responses and responses in which participants provided their e-mail but did not answer any items on the survey. SafeUT includes students ranging from grade school to college level; however, we excluded college-age students (above 12th grade and/or >18 years old) and conducted analyses only on grade school–age participants (12th grade and below) as we wanted to capture the unique characteristics and challenges presented in this particular group. Data about college-age students may be discussed in future publications. Analyses included descriptive statistics to assess the demographic characteristics of respondents (eg, age, grade, ethnicity, sex, gender identity, and sexual orientation), prior experiences with mental health services, frequency and level of difficulty presented by different barriers to accessing mental health services, and perception of participants of their SafeUT encounter. We also conducted paired *t* statistics (2-tailed) to evaluate whether participants experienced any significant changes in their presenting problems before vs after encounter. Finally, given that many participants endorsed having recent suicide attempts before the survey, we conducted exploratory analyses using χ^2^ tests to evaluate whether gender identity, sexuality, or current engagement in formal mental health services was associated with recent suicide attempts.

## Results

### Participants

After being given study information and entering their e-mail address, 349 youth provided assent to participate in the study. Of these participants, 60 did not answer any items and were thus removed from analyses. We also excluded 64 participants who endorsed being college students. Of the remaining 225 participants, 210 had at least completed the demographics section and indicated being in grade school (grades 4-12 and ages 9-18 years). See Figure S1, available online, for a flowchart of eligibility and completed survey sections. The distribution of race/ethnicity was consistent with the 2020 census for the state of Utah with 4.8% identifying as Asian, 1.2% as Black/African American, 9.5% as Hispanic/Latinx, 0.5% as Pacific Islander, and 85.7% as White either alone or in combination. Within this sample of 210 participants, 60 (28.6%) reported that their SafeUT encounter preceding the survey was their first interaction with SafeUT, 138 (65.7%) reported this was not their first SafeUT encounter, and 12 (5.7%) did not respond. Of note, less than half of the sample identified as being straight or heterosexual and more than a quarter identified as transgender, nonbinary, or gender nonconforming. Additional information is shown in Table 1. There were no differences in demographic characteristics (age, gender, sexual orientation, grade, race/ethnicity) in participants who discontinued early (following the demographics section) vs those who continued at least through the pre- and post-SafeUT encounter ratings.

**Table 1:** Demographic Characteristics of Participants

Characteristic	Sample (N = 210)	
	Mean	(SD)
Age, y	14.7	(1.93)
	**Median**	**[minimum, maximum]**
	15.0	[9.00-18.0]
	**n**	**(%)**
School grade		
4th	2	(0.6)
5th	3	(1.4)
6th	11	(5.2)
7th	24	(11.4)
8th	41	(19.5)
9th	28	(13.3)
10th	49	(23.3)
11th	29	(13.8)
12th	26	(12.4)
Gender		
Cisgender female	128	(61.0)
Cisgender male (cisgender)	23	(11.0)
Genderqueer/gender-nonconforming	30	(14.3)
Transgender female	4	(1.9)
Transgender male	5	(2.4)
Prefer to self-describe^a^	16	(7.6)
Prefer not to answer	4	(1.9)
Sexuality		
Asexual	10	(4.8)
Bisexual	45	(21.4)
Lesbian/gay	19	(9.0)
Pansexual	18	(8.6)
Queer	3	(1.4)
Questioning/unsure	18	(8.6)
Straight/heterosexual	83	(39.5)
Prefer to self-describe^b^	9	(4.3)
Prefer not to answer	5	(2.4)
Race/ethnicity^c^		
Asian	10	(4.8)
Black/African American	4	(1.9)
Hispanic/Latinx	20	(9.5)
Native American	5	(2.4)
Pacific Islander	1	(0.5)
White	180	(85.7)
Prefer to self-describe^d^	9	(4.3)
Prefer not to answer	8	(3.8)

**Note:**

a Responses included genderfluid = 2; demigirl = 3; nonbinary = 3; she/they pronouns = 3; agender = 2; nonbinary transmasculine = 1; agender with all neopronouns = 1; none = 1; she/her/he/him = 1.

b Responses included no label = 2; polyamory = 1; omnisexual = 1; nonbinary = 1; panromantic = 2; straight/bi-curious = 1; asexual pansexual = 1; aroace = 1; demisexual = 1.

c Participants could select multiple options for race/ethnicity.

d Responses included Middle Eastern = 1; White/Native American = 1; Irish-American = 1; Puerto Rican = 1; multiracial = 1; Brazilian Australian American = 1; unsure = 1; mix = 1.

### Mental Health Services and Support

Of 210 participants, before ever engaging with SafeUT, 95 (45.2%) reported having engaged in formal mental health services, including another crisis line, outpatient medication, outpatient therapy, inpatient hospitalization, day treatment, and intensive outpatient programming. When not including use of another crisis line, 83 (39.5%) accessed these formal services. A total of participants (43.3%) reported that they were currently receiving formal mental health supports at the time of completing the survey. This included outpatient therapy, outpatient medication management, day treatment, and intensive outpatient programming.

### Barriers to Mental Health Services

**Figure 1:**
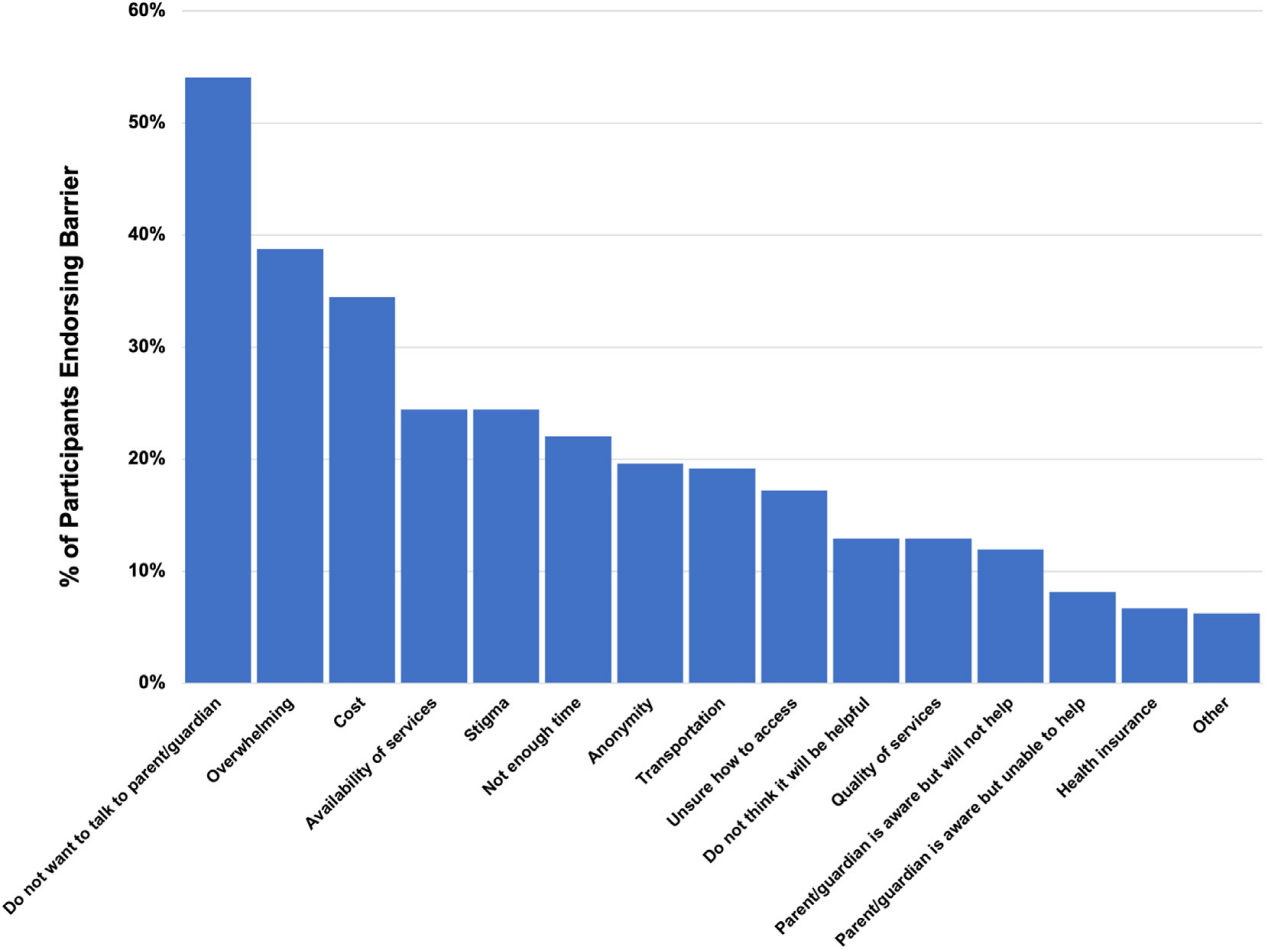
Barriers to Receiving Mental Health Services (n = 209) ***Note:****Graph showing frequency of participants endorsing that these circumstances acted as barriers to mental health service access.*

**Figure 2:**
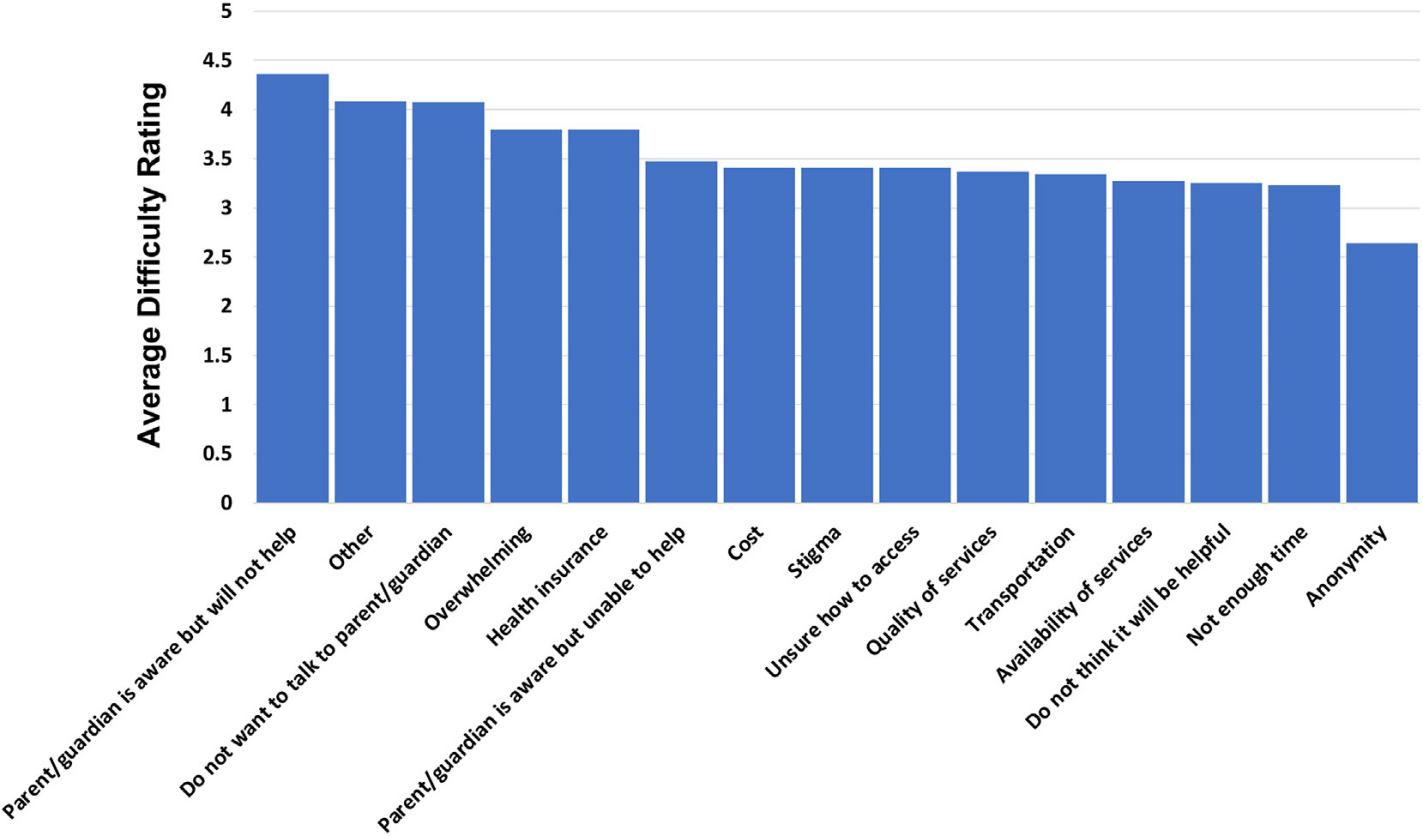
Difficulty Ratings for Mental Health Service Barriers ***Note:****Blue bars indicate average difficulty rating for each barrier. Average participant ratings of the level of difficulty each endorsed barrier presented on a scale of 1 (not at all difficult) to 5 (extremely difficult).*

Of 210 participants, 209 provided responses regarding barriers to receiving mental health services. The most endorsed barrier was “Do not want to talk to parent/guardian about it,” with 113 (54.1%) respondents identifying this as a barrier. Other top barriers included “It seems too overwhelming” (81/209; 38.8%) and “Cost” (72/209; 34.4%). Figure 1 depicts additional barriers and the frequency with which they were endorsed by participants. Themes of free text responses (n = 13) included distrust in adults, fear of consequences for disclosing SITBs, and perceived burdensomeness. Examples include “Myself, I don’t wanna be a burden on people,” “I am nervous about talking to others if I cut myself or something like that,” and “Difficulty trusting adults”. The barrier that was rated as being the most difficult was “Parent/guardian is aware but will not help,” with a mean score of 4.36. This was followed by “Other” (4.08) and “Do not want to talk to parent/guardian” (4.07). Figure 2 provides a depiction of difficulty ratings for barriers.

### Self-Injurious Thoughts and Behaviors

As shown in Table 2, the most common SITB experiences were thoughts of hurting oneself without suicidal intent (118/202; 58.4%), with ambivalent suicidal intent (114/202; 56.4%), and with suicidal intent (108/202; 53.5%). Altogether, this included 145 of 202 participants, or 71.8% of the sample. Of 202 participants, 102/ (50.5%) endorsed hurting themselves on purpose (regardless of suicidal intent), 85 (42.1%) endorsed hurting themselves on purpose without suicidal intent in the 2 weeks before the survey, 56 (27.7%) endorsed self-harm with ambivalent intent, and 37 (18.3%) reported having a suicide attempt during this 2-week period. Of the 37 participants who reported suicide attempts in the past 2 weeks, 30 (81%) also endorsed either nonsuicidal or ambivalent self-harm in the past 2 weeks.

**Table 2:** Participant Endorsement of Self-Injurious Thoughts and Behaviors (SITBs) in the 2 Weeks Before Taking the Survey

SITB item	Endorsements (n = 202 participants)		Mean intensity	Mean frequency
	n	(%)		
Thoughts of purposely hurting myself without wanting to die	118	58.42	3.34	3.02
Thoughts of purposely hurting myself with mixed feelings about dying	114	56.44	3.46	3.29
Thoughts of killing myself or suicide	108	53.47	3.3	3.14
Plans to purposely hurt myself without wanting to die	81	40.10	3.34	2.96
Plans to purposely hurt myself with mixed feelings about dying	74	36.63	3.32	3
Plans to kill myself	50	24.75	3.29	3.15
Communicated about hurting myself without wanting to die	60	29.70	3.02	2.36
Communicated about hurting myself with mixed feelings about dying	45	22.28	3.27	2.66
Communicated about killing myself	46	22.77	3.11	2.44
Hurt myself on purpose without wanting to die	85	42.08	3.29	2.77
Hurt myself on purpose with mixed feelings about dying	56	27.72	3.58	3.07
Attempted suicide with the intent to die	37	18.32	3.92	2.19

We followed up on the frequency of suicide attempts by conducting χ^2^ tests to determine whether there was a relation between suicide attempt in the past 2 weeks and characteristics such as gender, sexual orientation, or current formal treatment status. Due to small cell sizes for some gender identity responses when including only participants who responded to the suicide item, we converted gender to a binary variable with 2 levels: cisgender (n = 148) and transgender/nonbinary/gender nonconforming (n = 50). The relation between gender identity and suicide attempt in the prior 2 weeks was significant, indicating that participants who identified as transgender/nonbinary/gender nonconforming were more likely than cisgender participants to report a suicide attempt in the past 2 weeks (χ^2^_1_ = 4.7, *p* = .03). Similarly, we collapsed responses on reported sexuality to create a dichotomous variable with 2 levels: sexually minoritized (eg, asexual, bisexual, lesbian/gay, pansexual, queer; n = 115) and non–sexually minoritized (n = 81). There was a significant relation between sexual minoritized status and suicide attempt, with individuals who identified as being sexually minoritized more likely to report a suicide attempt in the past 2 weeks (χ^2^_1_ = 7.6, *p* = .006). There was no significant relation between current formal treatment status and suicide attempts (χ^2^_1_ = 3.0, *p* = .09. These results are not adjusted for multiple tests. However, the finding of increased suicide attempts among sexually minoritized participants stands when using a stringent Bonferroni-adjusted *p* threshold of .02.

### Reported Effectiveness

Regarding effectiveness of SafeUT, 182 participants provided responses. We observed a significant reduction in the intensity of all presenting concerns following the SafeUT encounter as shown in Table 3 (only reported for items endorsed by >5 participants). When using a Bonferroni-adjusted *p* value of .005, all presenting concerns had a significant reduction except for “I was thinking about hurting someone else” (*p* = .008), and “I wanted to hurt someone else” (*p* = .009).

**Table 3:** Change in Presenting Concern Intensity Following SafeUT Contact

Presenting Concern	Participants (n = 182)	Intensity Before SafeUT		Intensity After SafeUT		*t*	*p*
		Mean	(SD)	Mean	(SD)		
I was worrying or feeling sad	166	3.97	(1.01)	2.16	(1.14)	20.55	<.001
I was distressed about something that recently happened	128	4.19	(0.93)	2.3	(1.23)	17.65	<.001
I was thinking about hurting myself without wanting to die	62	3.82	(1.16)	2.1	(1.46)	9.5	<.001
I wanted to hurt myself without wanting to die	47	4	(1.02)	2.09	(1.35)	9.3	<.001
I was hurting myself on purpose without wanting to die	34	4.12	(1.23)	2.35	(1.69)	6.96	<.001
I was thinking about being dead/not alive anymore	78	3.94	(1.09)	2.18	(1.4)	11.03	<.001
I was thinking about killing myself	53^a^	3.75	(1.19)	2.25	(1.28)	8.92	<.001
I wanted to kill myself	46	3.83	(1.3)	2.28	(1.64)	7.04	<.001
I had done or was doing something to kill myself	14	3.71	(1.54)	2.79	(1.53)	3.79	.002
I was thinking about hurting someone else	9	3.33	(1.53)	1.44	(1.51)	3.41	.008
I wanted to hurt someone else	8	3.63	(1.19)	1.88	(1.25)	3.56	.009

**Note:**

a Of 53 participants who endorsed “I was thinking about killing myself,” 47 provided ratings. The 5 participants who did not provide ratings endorsed and provided ratings for “I was thinking about being dead/not alive anymore” (the item prior) and “I wanted to kill myself” (the item after).

Notably, there was a significant reduction in intensity for self-injurious behaviors and suicidal ideation from pre- to post-SafeUT interaction. This includes the following responses: “I was hurting myself on purpose without wanting to die” (pre mean [SD] = 4.12 [1.23], post = 2.35 [1.69]; *t*_33_ = 6.96, *p* < .001); “I was thinking about being dead/not alive anymore” (pre mean [SD] = 3.94 [1.09], post = 2.18 [1.4]; *t*_77_ = 11.03, *p* < .001); “I was thinking about killing myself” (pre mean [SD] = 3.75 [1.19], post = 2.25 [1.28]; *t*_47_ = 8.92, *p* < .001); “I wanted to kill myself” (pre mean [SD] = 3.83 [1.3], post = 2.28 [1.64]; *t*_45_ = 7.04, *p* < .001); and “I had done or was doing something to kill myself” (pre mean [SD] = 3.71 [1.8], post = 2.79 [1.53]; *t*_13_ = 3.79, *p* < .001).

When examining the presenting concerns that participants with suicide attempts indicated for their recent SafeUT contact, about 57% endorsed experiencing a suicide-related concern (thoughts of being dead, thoughts of killing self, wanting to kill self, or doing something to kill self). This highlights that despite experiencing a recent suicidal crisis, youth do not necessarily endorse seeking support for these concerns when contacting crisis services.

### Encounter Satisfaction

Regarding encounter satisfaction, of 182 participants who responded, 61.5% (112/182) reported feeling “very supported” (46/182), and 25.3% reported feeling “somewhat supported”; 10 participants reported feeling either “somewhat unsupported” and “very unsupported.” Of the 181 participants who provided a response, 52% reported feeling “very satisfied” (94/181). and 32% reported feeling “somewhat satisfied” (58/181); 4 participants reported feeling “somewhat dissatisfied,” and no participants reported feeling “very dissatisfied.” Of the 181 participants who responded, 89% (161/181) reported feeling the counselor either “usually” or “always” listened carefully to them, and 86.7% (157/181) reported the counselor “usually” or “always” explained things in a way they could understand. Of 180 participants who provided a response, 93.9% (169/180) reported that the counselor “usually” or “always” showed respect for what they had to say.

## Discussion

The present study provided an initial set of insights into the characteristics of youth who use a crisis service via an app. This included demographic characteristics, the frequency and intensity of SITBs, and experiences of participants with mental health service access and barriers. We also found promising results regarding their experiences and satisfaction with using the crisis app. More than half of the sample identified as being a member of the LGBTQ+ (lesbian, gay, bisexual, transgender, queer, and others) community, with a random sampling strategy based upon number of volleys in a conversation. This pattern is consistent with the large body of research that has documented higher risk for mental health crises among these youth, including self-injury and suicide.^10^, ^11^, ^12^, ^13^, ^14^, ^15^, ^16^, ^17^ Interestingly, nearly half of the sample reported currently receiving formal mental health services. This calls to question whether youth are openly working on SITBs with their providers and if they are receiving sufficient support in this area. Given that not wanting to talk to a parent or guardian was a large barrier for much of our sample, it is also possible that many youth do not feel comfortable discussing SITBs with their provider. In particular, individuals who experience SITBs often feel uncomfortable sharing these experiences with mental health professionals for fear of potential consequences such as inpatient hospitalization or even having their provider refuse to continue seeing them.^18^^,^^19^

The most common barriers reported by participants included not wanting to talk to their parent or guardian, feeling that seeking services is overwhelming and costly. Parent- and guardian-related barriers are especially critical and concerning, as many states, including Utah, require that a parent or guardian must first provide consent for their child to engage in substantive mental health services.^20^, ^21^, ^22^, ^23^ Our finding of parent-related barriers to mental health services is consistent with other recent research.^24^ This has important implications for future policy making; identifying no (or minimal) parental consent and low-cost treatment options may offer an easy way to reach many at-risk youth. Requirement of parental consent may be placing youth at increased risk for mental health difficulties including suicide.

Another concerning finding is that many youth reported engaging in some kind of self-injury, including suicide attempts, in the 2 weeks before completing the survey. While we would anticipate that a sizable proportion of our sample would report recent SITBs, given that we drew participants from a group of youth using a mental health text crisis service, this remains alarming. This is a relatively short time span within which nearly 20% of the sample made a suicide attempt. When examining the presenting concerns that participants with suicide attempts indicated for their recent SafeUT contact, only about 57% endorsed experiencing a suicide-related concern (thoughts of being dead, thoughts of killing self, wanting to kill self, or doing something to kill self). Moreover, the goal of the SafeUT app is to encourage reaching out before self-injury or a suicide attempt. This suggests considerable education and outreach may still be needed to promote earlier help seeking for self-harm.

While we are unable to determine the extent to which a suicide risk assessment was completed with these youth by SafeUT clinicians, it does highlight the importance of making a conscious effort to gather information about the severity and recency of prior attempts during crisis text interactions, which can be helpful (albeit limited) indicator of future risk.^25^^,^^26^ While efforts in the field to predict who will go on to attempt or be lost to suicide have been unsuccessful, gathering detailed information about suicide history can facilitate the application of more successful and timely safety planning.

There were widespread reductions in the intensity of most presenting concerns when comparing pre- vs post-SafeUT encounter ratings. When asked to rate the intensity of their experiences with SITBs before and after their encounter with a SafeUT clinician, participants rated the intensity of these experiences as being significantly lower following their conversation. These reductions included suicidal, nonsuicidal, and ambivalent thoughts and behaviors. While there was still an element of retrospective reporting given that they had to rate their pre-encounter intensity at the time of the survey (after the encounter), these results are consistent with other studies examining short-term effects of crisis service usage.^1^, ^2^, ^3^^,^^5^ Furthermore, participants were largely satisfied and endorsed feeling supported and respected during their encounter. This outcome may be facilitated through the training SafeUT clinicians receive. Presently, SafeUT training for clinicians incorporates themes from multiple theories, including basic crisis intervention theory, cognitive theory, and psychosocial transition model. Other studies have highlighted that particularly promising crisis line interventions include Applied Suicide Intervention Skills, which has been associated with callers feeling less depressed, suicidal, and overwhelmed and more hopeful relative to treatment as usual.^27^

The present study adds to a currently sparse area of research exploring use of mental health crisis services in youth. By developing a better understanding of the characteristics of the youth accessing these services, we can more mindfully train and prepare counselors providing these services. Given the high rate of service utilization by LGBTQ+ youth, it is imperative that counselors are well educated in working with this particular population in an effort to provide the most appropriate and empathic care that acknowledges the unique difficulties that these youth encounter. Suicidal ideation is associated with gender identity through the experience of minority stress, with the relation between minority stress and suicidal ideation being moderated by thwarted belongingness.^28^ This relation highlights the importance of fostering strong social support networks within this population, which can be facilitated in the context of crisis care.

Despite the value offered by this study, we are limited by sample size and the emphasis on preserving anonymity, which can lead to some challenges regarding incomplete or potentially inaccurate survey responses. We also had a predominantly White sample, further limiting our interpretations. This is especially important as suicide rates among ethnic and racial minorities have been increasing at an alarming rate, particularly among Black youth.^29^^,^^30^ Due to limitations in existing measures of SITBs, we also did not use an existing validated SITB measure. Instead, items were from a combination of existing measures in addition to expertise of 2 of the authors (M.W.S., S.E.C.). Further, a critical limitation of this study is that the survey opportunity was offered only to youth who had a conversation of a given length with the SafeUT clinician (10 volleys). While this was done to ensure that respondents had a substantial interaction on which to base their ratings, it inadvertently excludes a subset of youth who may initiate help seeking but may withdraw for any number of reasons. As one of these reasons may be due to dissatisfaction with their encounter with the SafeUT clinician, results indicating a favorable experience with SafeUT may be biased. Future work that includes this group is needed in an effort to reinforce the initiation of help seeking and to understand what the barriers are to continuing a crisis service encounter. Moreover, a majority of these users who engaged in the survey had used SafeUT previously. While this could be an unusual representation of the population of potential users, this may provide insight regarding the characteristics of individuals who repeatedly use crisis services.

To our knowledge, no other study has published results regarding the characteristics of users of services similar to SafeUT, aside from services provided at the national level. SafeUT differs from other crisis services in that the counselors are licensed mental health professionals with master’s-level education or higher. Future work would benefit from examining whether this level of training and licensure provides a significant advantage regarding the effectiveness of the service. In particular, the shortage of mental health providers places an additional strain on the availability of staff for crucial services such as crisis care, posing a significant limitation in expanding the SafeUT model at a national level. To address this limitation, additional initiatives would require more systematic training in evidence-based care in the context of crises as well as exploring and developing additional care models that use bachelor’s-level service delivery professionals. We have also considered using a step-down or step-up model for SafeUT that could incorporate peer certified counselors. However, determination of a stepped care model is nontrivial, and the variability in the reasons of SafeUT use among repeat users has precluded pursuing this option at this time.

Additional advantages of SafeUT beyond licensed clinicians include its integration within and bidirectional communication with the school and potentially greater ease of deploying a mobile crisis outreach team and related emergency responder teams in emergencies. However, a mobile crisis outreach team itself is a service available to very few communities at a national level. Significant investment is needed from all levels to improve infrastructure and accelerate workforce development to comprehensively and effectively address mental health crises in real time. Further, these findings strongly support the need for LGBTQ+-specific clinical training in the context of crisis care as this is a particularly vulnerable population. It is also imperative for research to focus on crisis service use and effectiveness among racial and ethnic minorities. While studies to date indicate that these services are helpful, we cannot assume that this is the most appropriate resource for all youth. Finally, results from this study highlight that parents pose a significant barrier to youth accessing mental health. Reasons may include fear of or discomfort with parent reactions, concern about availability of family resources, and unsuccessful attempts at seeking parental support in the past. Future work may address this barrier by providing parents with resources to help facilitate discussions about mental health. This may include providing parents with workshops to develop improved response strategies when children are experiencing emotional distress and potentially amending parental consent laws for mental health services.

## CRediT authorship contribution statement

**Mindy Westlund Schreiner:** Writing – review & editing, Writing – original draft, Visualization, Validation, Software, Resources, Project administration, Methodology, Investigation, Funding acquisition, Formal analysis, Data curation, Conceptualization. **Brian W. Farstead:** Writing – review & editing, Writing – original draft, Project administration, Data curation. **Myah Pazdera:** Writing – review & editing, Writing – original draft, Project administration, Data curation. **Amanda V. Bakian:** Writing – review & editing, Validation, Supervision, Methodology. **Brent M. Kious:** Writing – review & editing, Resources, Methodology. **Karen Manotas:** Conceptualization. **Sheila E. Crowell:** Writing – review & editing, Writing – original draft, Supervision, Conceptualization. **Erin A. Kaufman:** Writing – review & editing, Writing – original draft. **Scott A. Langenecker:** Writing – review & editing, Writing – original draft, Supervision, Resources, Methodology, Investigation, Funding acquisition, Conceptualization.
