# Supplementary figures and images for: Characteristics of Youth Crisis App Users: Mental Health Service Access and Barriers and Perceptions of Helpfulness
Source: JAACAP Open. 2024 Aug 28;3(3):421–30. doi: 10.1016/j.jaacop.2024.06.006 (PMC12414296; doi:10.1016/j.jaacop.2024.06.006)

| Figure S1: Flow of Participants from Consent to Analysis |
| --- |


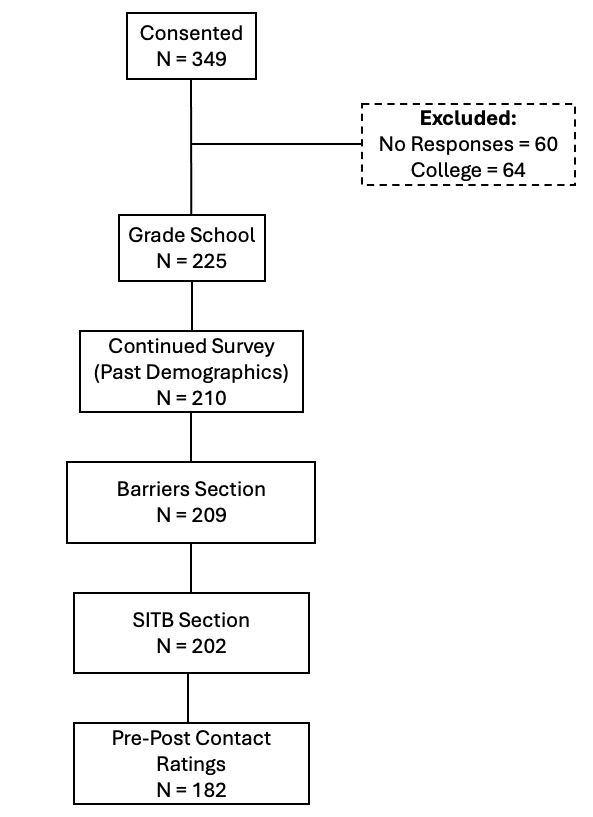

Supplement: Supplemental Figure 1 [file mmc1.docx]
