# Supplementary material for: Characteristics of Youth Crisis App Users: Mental Health Service Access and Barriers and Perceptions of Helpfulness
Source: JAACAP Open. 2024 Aug 28;3(3):421–30. doi: 10.1016/j.jaacop.2024.06.006 (PMC12414296; doi:10.1016/j.jaacop.2024.06.006)
Supplement: Supplemental 1 [file mmc2.docx]

**Supplement 1**

**Survey Questions**

The following are the questions that were presented to participants using REDCap. Note that many of the items used branching logic to reduce participant burden, in which certain items were only asked if preceding items were endorsed. For example, participants were only asked “How helpful were these services [Day treatment]?” if they endorsed having received Day treatment in the past.

First, we would like to learn a little bit about your background.

How old are you (in years)?

__________________________________

What grade are you in? (if summer, what grade did you most recently complete?)

- 1
- 2
- 3
- 4
- 5
- 6
- 7
- 8
- 9
- 10
- 11
- 12
- College/University Student

What zip code do you live in?

__________________________________

How would you describe your race/ethnicity? (check all that apply)

- Asian
- Black/African American
- Hispanic/Latinx
- Native American
- Pacific Islander
- White
- Prefer to self-describe [text field]
- Prefer not to answer

What is your ancestry, nationality, ethnic origin, or tribal affiliation? (e.g. Mexican, German, Sudanese, Ute, etc.)

__________________________________

(You can enter, None, Unsure if unsure or Prefer not to answer)

- What is your gender identity?
- Female (Cisgender)
- Genderqueer/Gender-nonconforming
- Male (Cisgender)
- Transgender male
- Transgender female
- Prefer to self-describe
- Prefer not to answer

Please describe your gender identity:

__________________________________

Which of the following best describes your sexual orientation?

- Asexual
- Bisexual
- Lesbian/Gay
- Pansexual
- Queer
- Questioning/Unsure
- Straight (Heterosexual)
- Prefer to self-describe
- Prefer not to answer

Please describe your sexual orientation:

__________________________________

What does your parent (e.g. mother, father) or legal guardian do for work? __________________________________

(Enter "Unsure" if you're not sure)

What is this parent/legal guardian's highest level of education?

- Less than high school
- High school or equivalent (e.g. GED)
- Some college, no degree
- Associate's degree (e.g. AA, AS)
- Bachelor's degree (e.g. BA, BS)
- Master's degree (e.g. MA, MS, MEd)
- Professional degree (e.g. MD, DDS, DVM)
- Doctorate degree (e.g. PhD, EdD)
- Unsure

Do you have another parent or legal guardian?

Yes

No

What does your second parent or legal guardian do for work? __________________________________

(Enter "Unsure" if you're not sure)

What is this parent/legal guardian's highest level of education?

- Less than high school
- High school or equivalent (e.g. GED)
- Some college, no degree
- Associate's degree (e.g. AA, AS)
- Bachelor's degree (e.g. BA, BS)
- Master's degree (e.g. MA, MS, MEd)
- Professional degree (e.g. MD, DDS, DVM)
- Doctorate degree (e.g. PhD, EdD)
- Unsure

Thank you for telling us some information about yourself. Next, we will ask you some questions about mental health.

Before you ever contacted SafeUT, which mental health resources or supports have you received? (check all that apply)

- Crisis line (other than SafeUT)
- Outpatient medication
- Outpatient therapy
- Day treatment (mental health treatment for around 8 hours most days per week at a hospital, clinic, or other facility)
- Inpatient hospitalization (staying overnight at the hospital due to mental health crisis)
- Intensive outpatient treatment (mental health treatment for a few hours per day for 3 days per week at a hospital, clinic, or other facility)
- Reaching out to a friend or family member
- Spiritual or religious advisor/counselor
- Self-help resources (readings, books, apps, forums)
- Mentorship programs (e.g. Big Brother/Big Sister)
- Other
- None of the above

Please Describe:

__________________________________

How helpful were these services [Crisis line (other than SafeUT)]?

- Very Helpful
- Somewhat Helpful
- Somewhat Unhelpful
- Very Unhelpful
- Unsure

How helpful were these services [Outpatient medication]?

- Very Helpful
- Somewhat Helpful
- Somewhat Unhelpful
- Very Unhelpful
- Unsure

How helpful were these services [Outpatient therapy]?

- Very Helpful
- Somewhat Helpful
- Somewhat Unhelpful
- Very Unhelpful
- Unsure

How helpful were these services [Day treatment]?

- Very Helpful
- Somewhat Helpful
- Somewhat Unhelpful
- Very Unhelpful
- Unsure

How helpful were these services [Inpatient hospitalization]?

- Very Helpful
- Somewhat Helpful
- Somewhat Unhelpful
- Very Unhelpful
- Unsure

How helpful were these services [Intensive outpatient treatment ]?

- Very Helpful
- Somewhat Helpful
- Somewhat Unhelpful
- Very Unhelpful
- Unsure

How helpful were these services [Reaching out to a friend or family member]?

- Very Helpful
- Somewhat Helpful
- Somewhat Unhelpful
- Very Unhelpful
- Unsure

How helpful were these services [Spiritual or religious advisor/counselor]?

- Very Helpful
- Somewhat Helpful
- Somewhat Unhelpful
- Very Unhelpful
- Unsure

How helpful were these services [Self-help resources]?

- Very Helpful
- Somewhat Helpful
- Somewhat Unhelpful
- Very Unhelpful
- Unsure

How helpful were these services [Mentorship programs]?

- Very Helpful
- Somewhat Helpful
- Somewhat Unhelpful
- Very Unhelpful
- Unsure

How helpful were these services [Other]?

- Very Helpful
- Somewhat Helpful
- Somewhat Unhelpful
- Very Unhelpful
- Unsure

Since the first time you contacted SafeUT, which mental health resources or supports have you received? (check all that apply)

- Crisis line (other than SafeUT)
- Outpatient medication
- Outpatient therapy
- Day treatment (mental health treatment for around 8 hours most days per week at a hospital, clinic, or other facility)
- Inpatient hospitalization (staying overnight at the hospital due to mental health crisis)
- Intensive outpatient treatment (mental health treatment for a few hours per day for 3 days per week at a hospital, clinic, or other facility)
- Reaching out to a friend or family member
- Spiritual or religious advisor/counselor
- Self-help resources (readings, books, apps, forums)
- Mentorship programs (e.g. Big Brother/Big Sister)
- Other
- None of the above/This was my first time contacting SafeUT
- None of the above

Please Describe:

__________________________________

How helpful were these services [Crisis line (other than SafeUT)]?

- Very Helpful
- Somewhat Helpful
- Somewhat Unhelpful
- Very Unhelpful
- Unsure

How helpful were these services [Outpatient medication]?

- Very Helpful
- Somewhat Helpful
- Somewhat Unhelpful
- Very Unhelpful
- Unsure

How helpful were these services [Outpatient therapy]?

- Very Helpful
- Somewhat Helpful
- Somewhat Unhelpful
- Very Unhelpful
- Unsure

How helpful were these services [Day treatment]?

- Very Helpful
- Somewhat Helpful
- Somewhat Unhelpful
- Very Unhelpful
- Unsure

How helpful were these services [Inpatient hospitalization]?

- Very Helpful
- Somewhat Helpful
- Somewhat Unhelpful
- Very Unhelpful
- Unsure

How helpful were these services [Intensive outpatient treatment]?

- Very Helpful
- Somewhat Helpful
- Somewhat Unhelpful
- Very Unhelpful
- Unsure

How helpful were these services [Reaching out to a friend or family member]?

- Very Helpful
- Somewhat Helpful
- Somewhat Unhelpful
- Very Unhelpful
- Unsure

How helpful were these services [Spiritual or religious advisor/counselor]?

- Very Helpful
- Somewhat Helpful
- Somewhat Unhelpful
- Very Unhelpful
- Unsure

How helpful were these services [Self-help resources]?

- Very Helpful
- Somewhat Helpful
- Somewhat Unhelpful
- Very Unhelpful
- Unsure

How helpful were these services [Mentorship programs]?

- Very Helpful
- Somewhat Helpful
- Somewhat Unhelpful
- Very Unhelpful
- Unsure

How helpful were these services [Other]?

- Very Helpful
- Somewhat Helpful
- Somewhat Unhelpful
- Very Unhelpful
- Unsure

Which mental health services do you currently receive? (check all that apply)

- Outpatient medication
- Outpatient therapy
- Day treatment (mental health treatment for around 8 hours most days per week at a hospital, clinic, or other facility)
- Intensive outpatient treatment (mental health treatment for a few hours per day for 3 days per week at a hospital, clinic, or other facility)
- Reaching out to a friend or family member
- Spiritual or religious advisor/counselor
- Self-help resources (readings, books, apps, forums)
- Mentorship programs (e.g. Big Brother/Big Sister)
- Other
- None of the above

Please Describe:

__________________________________

How helpful were these services [Outpatient medication]?

- Very Helpful
- Somewhat Helpful
- Somewhat Unhelpful
- Very Unhelpful
- Unsure

How helpful were these services [Outpatient therapy]?

- Very Helpful
- Somewhat Helpful
- Somewhat Unhelpful
- Very Unhelpful
- Unsure

How helpful were these services [Day treatment]?

- Very Helpful
- Somewhat Helpful
- Somewhat Unhelpful
- Very Unhelpful
- Unsure

How helpful were these services [Intensive outpatient treatment]?

- Very Helpful
- Somewhat Helpful
- Somewhat Unhelpful
- Very Unhelpful
- Unsure

How helpful were these services [Reaching out to a friend or family member]?

- Very Helpful
- Somewhat Helpful
- Somewhat Unhelpful
- Very Unhelpful
- Unsure

How helpful were these services [Spiritual or religious advisor/counselor]?

- Very Helpful
- Somewhat Helpful
- Somewhat Unhelpful
- Very Unhelpful
- Unsure

How helpful were these services [Self-help resources]?

- Very Helpful
- Somewhat Helpful
- Somewhat Unhelpful
- Very Unhelpful
- Unsure

How helpful were these services [Mentorship programs]?

- Very Helpful
- Somewhat Helpful
- Somewhat Unhelpful
- Very Unhelpful
- Unsure

How helpful were these services [Other]?

- Very Helpful
- Somewhat Helpful
- Somewhat Unhelpful
- Very Unhelpful
- Unsure

Do any of these problems make it hard for you to get mental health help? (select all that apply) Availability of therapists or other service providers

- Cost
- No health insurance/mental health not covered
- Transportation
- Unsure how to access services
- Do not want to talk to parent/guardian about it
- Parent/guardian is aware and unable to help
- Parent/guardian is aware, but will not help
- Prefer to stay anonymous
- Concern about stigma or shame
- Not enough time
- It seems too overwhelming
- Do not think it would be helpful
- Quality of service provided by therapists or other service providers
- Other
- I have not experienced any difficulties

If Other, please describe:

__________________________________

How difficult does the following problem make getting mental health help? Availability of therapists or other service providers

---------------------------------------------------------------------

Not at all difficult Extremely difficult

(Place a mark on the scale above)

How difficult does the following problem make getting mental health help? Cost

---------------------------------------------------------------------

Not at all difficult Extremely difficult

(Place a mark on the scale above)

How difficult does the following problem make getting mental health help? No health insurance/mental health not covered

---------------------------------------------------------------------

Not at all difficult Extremely difficult

(Place a mark on the scale above)

How difficult does the following problem make getting mental health help? Transportation

---------------------------------------------------------------------

Not at all difficult Extremely difficult

(Place a mark on the scale above)

How difficult does the following problem make getting mental health help? Unsure how to access services

---------------------------------------------------------------------

Not at all difficult Extremely difficult

(Place a mark on the scale above)

How difficult does the following problem make getting mental health help? Do not want to talk to parent/guardian about it

---------------------------------------------------------------------

Not at all difficult Extremely difficult

(Place a mark on the scale above)

How difficult does the following problem make getting mental health help? Parent/guardian is aware and unable to help

---------------------------------------------------------------------

Not at all difficult Extremely difficult

(Place a mark on the scale above)

How difficult does the following problem make getting mental health help? Parent/guardian is aware, not help

---------------------------------------------------------------------

Not at all difficult Extremely difficult

(Place a mark on the scale above)

How difficult does the following problem make getting mental health help? Prefer to stay anonymous

---------------------------------------------------------------------

Not at all difficult Extremely difficult

(Place a mark on the scale above)

How difficult does the following problem make getting mental health help? Concern about stigma or shame

---------------------------------------------------------------------

Not at all difficult Extremely difficult

(Place a mark on the scale above)

How difficult does the following problem make getting mental health help? Not enough time ---------------------------------------------------------------------

Not at all difficult Extremely difficult

(Place a mark on the scale above)

How difficult does the following problem make getting mental health help? It seems too overwhelming

---------------------------------------------------------------------

Not at all difficult Extremely difficult

(Place a mark on the scale above)

How difficult does the following problem make getting mental health help? Do not think it would be helpful

---------------------------------------------------------------------

Not at all difficult Extremely difficult

(Place a mark on the scale above)

How difficult does the following problem make getting mental health help? Quality of service provided by therapists or other service providers

---------------------------------------------------------------------

Not at all difficult Extremely difficult

(Place a mark on the scale above)

How difficult does the following problem make getting mental health help? Other

---------------------------------------------------------------------

Not at all difficult Extremely difficult

(Place a mark on the scale above)

Next, we would like some information about difficulties some people have. Have you

experienced any of the following in the past two weeks?

***Please read these carefully, as the questions are similar, but are all slightly different.***

Thoughts of purposely hurting myself without wanting to die

Yes No

If Yes:

How intense were these thoughts?

---------------------------------------------------------------------

Not at all intense Somewhat intense Very intense

(Place a mark on the scale above)

How frequent were these thoughts?

---------------------------------------------------------------------

Not at all frequent Somewhat frequent Very frequent

(Place a mark on the scale above)

Thoughts of purposely hurting myself with mixed feelings about dying

Yes No

If Yes:

How intense were these thoughts?

---------------------------------------------------------------------

Not at all intense Somewhat intense Very intense

(Place a mark on the scale above)

How frequent were these thoughts?

---------------------------------------------------------------------

Not at all frequent Somewhat frequent Very frequent

(Place a mark on the scale above)

Thoughts of killing myself or suicide

Yes No

If Yes:

How intense were these thoughts?

---------------------------------------------------------------------

Not at all intense Somewhat intense Very intense

(Place a mark on the scale above)

How frequent were these thoughts?

---------------------------------------------------------------------

Not at all frequent Somewhat frequent Very frequent

(Place a mark on the scale above)

Plans to purposely hurt myself without wanting to die

Yes No

If Yes:

How intense were these plans?

---------------------------------------------------------------------

Not at all intense Somewhat intense Very intense

(Place a mark on the scale above)

How frequent were these plans?

---------------------------------------------------------------------

Not at all frequent Somewhat frequent Very frequent

(Place a mark on the scale above)

Plans to purposely hurt myself with mixed feelings about dying

Yes No

If Yes:

How intense were these plans?

---------------------------------------------------------------------

Not at all intense Somewhat intense Very intense

(Place a mark on the scale above)

How frequent were these plans?

---------------------------------------------------------------------

Not at all frequent Somewhat frequent Very frequent

(Place a mark on the scale above)

Plans to kill myself

Yes No

If Yes:

How intense were these plans?

---------------------------------------------------------------------

Not at all intense Somewhat intense Very intense

(Place a mark on the scale above)

How frequent were these plans?

---------------------------------------------------------------------

Not at all frequent Somewhat frequent Very frequent

(Place a mark on the scale above)

Communicated about hurting myself without wanting to die

Yes No

If Yes:

How intense were these communications?

---------------------------------------------------------------------

Not at all intense Somewhat intense Very intense

(Place a mark on the scale above)

How frequent were these communications?

---------------------------------------------------------------------

Not at all frequent Somewhat frequent Very frequent

(Place a mark on the scale above)

Communicated about hurting myself with mixed feelings about dying

Yes No

If Yes:

How intense were these communications?

---------------------------------------------------------------------

Not at all intense Somewhat intense Very intense

(Place a mark on the scale above)

How frequent were these communications?

---------------------------------------------------------------------

Not at all frequent Somewhat frequent Very frequent

(Place a mark on the scale above)

Communicated about killing myself

Yes No

If Yes:

How intense were these communications?

---------------------------------------------------------------------

Not at all intense Somewhat intense Very intense

(Place a mark on the scale above)

How frequent were these communications?

---------------------------------------------------------------------

Not at all frequent Somewhat frequent Very frequent

(Place a mark on the scale above)

Hurt myself on purpose without wanting to die

Yes No

If Yes:

How intense was this experience?

---------------------------------------------------------------------

Not at all intense Somewhat intense Very intense

(Place a mark on the scale above)

How frequent was this experience?

---------------------------------------------------------------------

Not at all frequent Somewhat frequent Very frequent

(Place a mark on the scale above)

Hurt myself on purpose with mixed feelings about dying

Yes No

If Yes:

How intense was this experience?

---------------------------------------------------------------------

Not at all intense Somewhat intense Very intense

(Place a mark on the scale above)

How frequent was this experience?

---------------------------------------------------------------------

Not at all frequent Somewhat frequent Very frequent

(Place a mark on the scale above)

Attempted suicide with the intent to die

Yes No

If Yes:

How intense was this experience?

---------------------------------------------------------------------

Not at all intense Somewhat intense Very intense

(Place a mark on the scale above)

How frequent was this experience?

---------------------------------------------------------------------

Not at all frequent Somewhat frequent Very frequent

(Place a mark on the scale above)

Now we will ask some questions about your use of SafeUT

Was this the first time you contacted SafeUT?

Yes No

Including this time, how many times have you contacted SafeUT? (please give your best guess)

- 1
- 2
- 3
- 4
- 5
- 6
- 7
- 8
- 9
- 10
- 11
- 12
- 13
- 14
- 15
- 16
- 17
- 18
- 19
- 20+

The following questions are about your experiences with SafeUT in the past

Considering the concerns that you had when you contacted SafeUT for the first time, please

answer the following set of questions.

When you contacted SafeUT in the past, how supported did you feel by the SafeUT counselor?

- Very unsupported
- Somewhat unsupported
- Neither unsupported nor supported
- Somewhat supported
- Very supported
- Unsure

When you contacted SafeUT in the past, how satisfied were you with how your concern was handled?

- Very dissatisfied
- Somewhat dissatisfied
- Neither satisfied nor dissatisfied
- Somewhat satisfied
- Very satisfied
- Unsure

When you contacted SafeUT in the past, how often did you feel the counselor listened carefully to you?

- Never
- Rarely
- Sometimes
- Usually
- Always

When you contacted SafeUT in the past, how often did you feel the counselor explained things in a way you could understand?

- Never
- Rarely
- Sometimes
- Usually
- Always

When you contacted SafeUT in the past, how often did you feel the counselor showed respect for what you had to say?

- Never
- Rarely
- Sometimes
- Usually
- Always

When you contacted SafeUT in the past, how many resources or supports were provided by the SafeUT counselor?

- A number of options
- A few options
- Almost no options
- No options at all

Have you followed through with any resources or supports provided by a SafeUT counselor in the past?

Yes No

Which of the following resources or supports did you seek because of your interaction with SafeUT? (check all that apply)

- Day treatment (mental health treatment for around 8 hours most days per week at a hospital, clinic, or other facility)
- Inpatient hospitalization (staying overnight at the hospital due to mental health crisis)
- Intensive outpatient treatment (mental health treatment for a few hours per day for 3 days per week at a hospital, clinic, or other facility)
- Mentorship programs (e.g. Big Brother/Big Sister)
- Self-help resources (readings, books, apps, forums)
- Outpatient medication
- Outpatient therapy
- Reaching out to a friend or family member
- Spiritual or religious advisor/counselor
- Other
- None of the above

If Other, please describe:

__________________________________

When you contacted SafeUT in the past, how motivated were you to seek mental health services?

---------------------------------------------------------------------

Not at all motivated Very motivated

(Place a mark on the scale above)

In the past, did the SafeUT counselor help you make a plan to get mental health care? (check all that apply)

- Yes, and I got help
- Yes, but I did not get help in the end
- Yes, but I changed my mind
- Yes, but it was not helpful
- No
- Other

If Other, please describe:

__________________________________

When you tried to get mental health support, did you run into problems that you or the SafeUT counselor did not expect?

Yes No

If Yes, please describe:

__________________________________

Based on your experience with SafeUT in the past, how much has SafeUT made you more or less likely to seek additional mental health services?

---------------------------------------------------------------------

Much less likely Made no difference Much more likely

(Place a mark on the scale above)

Please rate how intense these concerns were for you when you contacted SafeUT this time. We will ask a set of slightly different questions. Please answer all that are relevant to this specific time that you contacted SafeUT.

I was worrying or feeling sad

Yes No

If Yes:

How intense were these feelings before you contacted SafeUT?

---------------------------------------------------------------------

Not at all intense Somewhat intense Very intense

(Place a mark on the scale above)

How intense were these feelings after you contacted SafeUT?

---------------------------------------------------------------------

Not at all intense Somewhat intense Very intense

(Place a mark on the scale above)

I was distressed about something that recently happened

Yes No

If Yes:

How intense were these feelings before you contacted SafeUT?

---------------------------------------------------------------------

Not at all intense Somewhat intense Very intense

(Place a mark on the scale above)

How intense were these feelings after you contacted SafeUT?

---------------------------------------------------------------------

Not at all intense Somewhat intense Very intense

(Place a mark on the scale above)

I was thinking about hurting myself without wanting to die

If Yes:

How intense were these thoughts before you contacted SafeUT?

---------------------------------------------------------------------

Not at all intense Somewhat intense Very intense

(Place a mark on the scale above)

How intense were these thoughts after you contacted SafeUT?

---------------------------------------------------------------------

Not at all intense Somewhat intense Very intense

(Place a mark on the scale above)

I wanted to hurt myself without wanting to die

Yes No

If Yes:

How intense were these thoughts before you contacted SafeUT?

---------------------------------------------------------------------

Not at all intense Somewhat intense Very intense

(Place a mark on the scale above)

How intense were these thoughts after you contacted SafeUT?

---------------------------------------------------------------------

Not at all intense Somewhat intense Very intense

(Place a mark on the scale above)

I was hurting myself on purpose without wanting to die

Yes No

If Yes:

How intense was this experience before you contacted SafeUT?

---------------------------------------------------------------------

Not at all intense Somewhat intense Very intense

(Place a mark on the scale above)

How intense was this experience after you contacted SafeUT?

---------------------------------------------------------------------

Not at all intense Somewhat intense Very intense

(Place a mark on the scale above)

I was thinking about being dead/not alive anymore

Yes No

If Yes:

How intense were these thoughts before you contacted SafeUT?

---------------------------------------------------------------------

Not at all intense Somewhat intense Very intense

(Place a mark on the scale above)

How intense were these thoughts after you contacted SafeUT?

---------------------------------------------------------------------

Not at all intense Somewhat intense Very intense

(Place a mark on the scale above)

I was thinking about killing myself

Yes No

If Yes:

How intense were these thoughts before you contacted SafeUT?

---------------------------------------------------------------------

Not at all intense Somewhat intense Very intense

(Place a mark on the scale above)

How intense were these thoughts after you contacted SafeUT?

---------------------------------------------------------------------

Not at all intense Somewhat intense Very intense

(Place a mark on the scale above)

I wanted to kill myself

Yes No

If Yes:

How intense were these thoughts before you contacted SafeUT?

---------------------------------------------------------------------

Not at all intense Somewhat intense Very intense

(Place a mark on the scale above)

How intense were these thoughts after you contacted SafeUT?

---------------------------------------------------------------------

Not at all intense Somewhat intense Very intense

(Place a mark on the scale above)

I had done or was doing something to kill myself

Yes No

If Yes:

How intense was this experience before you contacted SafeUT?

---------------------------------------------------------------------

Not at all intense Somewhat intense Very intense

(Place a mark on the scale above)

How intense was this experience after you contacted SafeUT?

---------------------------------------------------------------------

Not at all intense Somewhat intense Very intense

(Place a mark on the scale above)

I was thinking about hurting someone else

Yes No

If Yes:

How intense were these thoughts before you contacted SafeUT?

---------------------------------------------------------------------

Not at all intense Somewhat intense Very intense

(Place a mark on the scale above)

How intense were these thoughts after you contacted SafeUT?

---------------------------------------------------------------------

Not at all intense Somewhat intense Very intense

(Place a mark on the scale above)

I wanted to hurt someone else

Yes No

If Yes:

How intense were these thoughts before you contacted SafeUT?

---------------------------------------------------------------------

Not at all intense Somewhat intense Very intense

(Place a mark on the scale above)

How intense were these thoughts after you contacted SafeUT?

---------------------------------------------------------------------

Not at all intense Somewhat intense Very intense

(Place a mark on the scale above)

I was planning on hurting someone else

Yes No

If Yes:

How intense were these thoughts before you contacted SafeUT?

---------------------------------------------------------------------

Not at all intense Somewhat intense Very intense

(Place a mark on the scale above)

How intense were these thoughts after you contacted SafeUT?

---------------------------------------------------------------------

Not at all intense Somewhat intense Very intense

(Place a mark on the scale above)

I had hurt or was hurting someone else

Yes No

If Yes:

How intense was this experience before you contacted SafeUT?

---------------------------------------------------------------------

Not at all intense Somewhat intense Very intense

(Place a mark on the scale above)

How intense was this experience after you contacted SafeUT?

---------------------------------------------------------------------

Not at all intense Somewhat intense Very intense

(Place a mark on the scale above)

How supported did you feel by the SafeUT counselor?

- Very unsupported
- Somewhat unsupported
- Neither unsupported nor supported
- Somewhat supported
- Very supported
- Unsure

How satisfied are you with how your concern was handled?

- Very dissatisfied
- Somewhat dissatisfied
- Neither satisfied nor dissatisfied
- Somewhat satisfied
- Very satisfied
- Unsure

How often do you feel the counselor listened carefully to you?

- Never
- Rarely
- Sometimes
- Usually
- Always

How often do you feel the counselor explained things in a way you could understand?

- Never
- Rarely
- Sometimes
- Usually
- Always

How often do you feel the counselor showed respect for what you had to say?

- Never
- Rarely
- Sometimes
- Usually
- Always

How many resources or supports were provided by the SafeUT counselor this time?

- A number of options
- A few options
- Almost no options
- No options at all

Which resources or supports did the SafeUT counselor provide? (check all that apply)

- Day treatment (mental health treatment for around 8 hours most days per week at a hospital, clinic, or other facility)
- Inpatient hospitalization (staying overnight at the hospital due to mental health crisis)
- Intensive outpatient treatment (mental health treatment for a few hours per day for 3 days per week at a hospital, clinic, or other facility)
- Mentorship programs (e.g. Big Brother/Big Sister)
- Self-help resources (readings, books, apps, forums)
- Outpatient medication
- Outpatient therapy
- Reaching out to a friend or family member
- Spiritual or religious advisor/counselor
- Other
- None of the above

If Other, please describe: __________________________________

Did the SafeUT counselor help you make a plan to get additional mental health care? (check all that apply)

- Yes, and I got help
- Yes, but I did not get help in the end
- Yes, but I changed my mind
- Yes, but it was not helpful
- No
- Other

If Other, please describe:__________________________________

How likely will you use any of these resources in the near future?

---------------------------------------------------------------------

Very unlikely Somewhat likely Very likely

(Place a mark on the scale above)

How motivated are you to continue current or seek additional mental health services right now?

---------------------------------------------------------------------

Not at all motivated Somewhat motivated Very motivated

(Place a mark on the scale above)

The next questions are you are doing during the COVID-19 pandemic.

How well have you been coping with the life disruptions caused by the pandemic?

---------------------------------------------------------------------

Not at all well Very well

(Place a mark on the scale above)

How well have you been coping with the uncertainty

related to the pandemic?

---------------------------------------------------------------------

Not at all well Very well

(Place a mark on the scale above)
